# 1-(4-Meth­oxy­phen­yl)-4-(3-nitro­phen­yl)-3-phen­oxy­azetidin-2-one

**DOI:** 10.1107/S1600536811003382

**Published:** 2011-01-29

**Authors:** Zeliha Baktır, Mehmet Akkurt, Aliasghar Jarrahpour, Roghaye Heiran

**Affiliations:** a Department of Physics, Faculty of Sciences, Erciyes University, 38039 Kayseri, Turkey; b Department of Chemistry, College of Sciences, Shiraz University, 71454 Shiraz, Iran

## Abstract

In the title compound, C_22_H_18_N_2_O_5_, the four-membered β-lactam ring is nearly planar, with a maximum deviation of 0.023 (2) Å for the N atom, and has long C—C distances of 1.525 (5) and 1.571 (5) Å. The mean plane of this group makes dihedral angles of 11.61 (19), 74.5 (2) and 72.3 (2)° with three aromatic rings. An intra­molecular C—H⋯O hydrogen bond occurs. The packing of the mol­ecules in the crystal structure is governed mainly by inter­molecular C—H⋯O hydrogen-bonding and C—H⋯π stacking inter­actions. Furthermore, a π–π inter­action [centroid–centroid distance = 3.6129 (19) Å] helps to stabilize the crystal structure.

## Related literature

For general background to β-lactams, see: Jubie *et al.* (2009 ▶); Mehta *et al.* (2010 ▶); Vatmurge *et al.* (2008 ▶); Von Nussbaum *et al.* (2006 ▶). For related structures, see: Akkurt *et al.* (2006 ▶); Ercan *et al.* (1996*a* ▶,*b* ▶); Kabak *et al.* (1999 ▶).
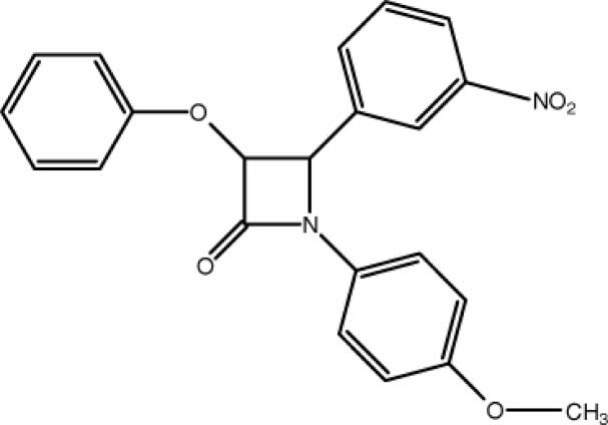


## Experimental

### 

#### Crystal data

C_22_H_18_N_2_O_5_
*M* *_r_* = 390.38
Triclinic, 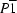

*a* = 7.7934 (4) Å
*b* = 11.2813 (3) Å
*c* = 11.8818 (2) Å
α = 77.771 (4)°
β = 80.948 (5)°
γ = 71.052 (4)°
*V* = 961.18 (6) Å^3^
*Z* = 2
Mo *K*α radiation
μ = 0.10 mm^−1^
*T* = 294 K
0.20 × 0.20 × 0.20 mm

#### Data collection

Rigaku R-AXIS RAPID-S diffractometer
Absorption correction: multi-scan (*SORTAV*; Blessing, 1995 ▶) *T*_min_ = 0.981, *T*_max_ = 0.981
3928 measured reflections
3928 independent reflections
1872 reflections with *I* > 2σ(*I*)
*R*_int_ = 0.104

#### Refinement

*R*[*F* ^2^ > 2σ(*F* ^2^)] = 0.059
*wR*(*F* ^2^) = 0.155
*S* = 1.01
3927 reflections
265 parameters
H-atom parameters constrained
Δρ_max_ = 0.14 e Å^−3^
Δρ_min_ = −0.15 e Å^−3^

### 

Data collection: *CrystalClear* (Rigaku/MSC, 2005 ▶); cell refinement: *CrystalClear*; data reduction: *CrystalClear*; program(s) used to solve structure: *SIR97* (Altomare *et al.*, 1999 ▶); program(s) used to refine structure: *SHELXL97* (Sheldrick, 2008 ▶); molecular graphics: *ORTEP-3 for Windows* (Farrugia, 1997 ▶); software used to prepare material for publication: *WinGX* (Farrugia, 1999 ▶).

## Supplementary Material

Crystal structure: contains datablocks global, I. DOI: 10.1107/S1600536811003382/bv2175sup1.cif

Structure factors: contains datablocks I. DOI: 10.1107/S1600536811003382/bv2175Isup2.hkl

Additional supplementary materials:  crystallographic information; 3D view; checkCIF report

## Figures and Tables

**Table 1:** Hydrogen-bond geometry (Å, °) *Cg*2 and *Cg*4 are the centroids of the C1–C6 and C17–C22 benzene rings, respectively.

*D*—H⋯*A*	*D*—H	H⋯*A*	*D*⋯*A*	*D*—H⋯*A*
C1—H1⋯O2	0.93	2.53	3.125 (4)	122
C2—H2⋯O2^i^	0.93	2.59	3.464 (4)	156
C16—H16⋯O4^ii^	0.93	2.52	3.164 (5)	126
C4—H4⋯*Cg*4^iii^	0.93	2.89	3.716 (4)	149
C9—H9⋯*Cg*2^iv^	0.98	2.55	3.463 (4)	154

Symmetry codes: (i) 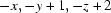; (ii) 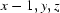; (iii) 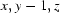; (iv) 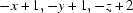.

**Fig. 1.**
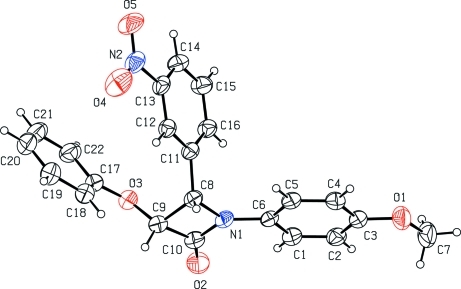
The title molecule with the atom numbering scheme. Displacement ellipsoids for non-H atoms are drawn at the 30% probability level.

**Fig. 2.**
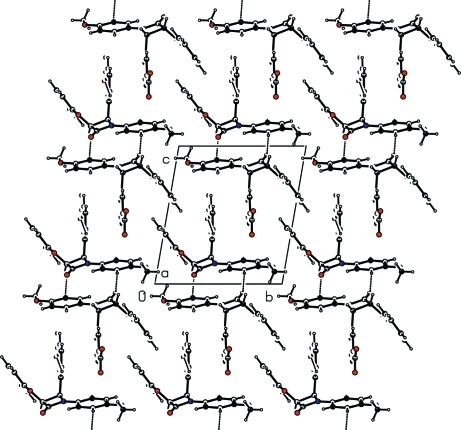
The packing diagram and the hydrogen bonding interactions of (I), viewing down the a axis.

## supplementary crystallographic information

### Comment

Azetidine-2-ones (β-lactams) were the first group of antibacterial natural products introduced as a therapeutic treatment of bacterial infections (Von Nussbaum *et al.*. 2006). The biological activity of  β-lactams is mostly believed to be associated with the chemical reactivity of their β-lactam ring and on its substituents, especially at the nitrogen of the 2-azetidinone ring (Mehta *et al.* 2010). p-Anisidine derivatives have been found to be biologically interesting for many years (Jubie *et al.* 2009). Continuous change in the structures of known active compounds and the preparation of new types (Vatmurge *et al.* 2008) has been forced by the development of bacterial resistance to known compounds (Vatmurge *et al*. 2008).

In the title compound (I), (Fig. 1), the β-lactam ring (N1/C8–C10) is nearly planar, with a maximum deviation of 0.023 (2)Å for N1. The bond lengths in the β-lactam ring are comparable with those found in previous similar studies (Akkurt *et al.* 2006; Ercan *et al.*, 1996*a*,*b*; Kabak *et al.*, 1999).

Its mean plane makes dihedral angles of 11.61 (19), 74.5 (2) and 72.3 (2)°, respectively, with three aromatic rings (C1–C6), (C11–C16) and (C17–C22). The details of the dihedral angles between the planes of the rings are given in Table 2.

A weak intramolecular C—H···O hydrogen bond contributes to the stability of the molecular configuration (Table 1). The crystal structure is stabilized by intermolecular C—H···O hydrogen-bonding (Table 1, Fig. 2) and C—H···π stacking interactions (Table 1). Furthermore, a π-π interaction [*Cg*1···*Cg*3(*x*, *y*, *z*) = 3.6129 (19) Å, *Cg*1 and *Cg*3 are the centroids of the N1/C8–C10 β-lactam ring and the C11–CC16 benzene rings, respectively] helps to stabilize the crystal structure.

### Experimental

A mixture of *N*-(3-nitrobenzylidene)-4-methoxybenzeneamine (1.28 g, 5.00 mmol) and triethylamine (2.53 g, 25.00 mmol), phenoxyacetic acid (1.14 g, 7.50 mmol) and tosyl chloride (1.43 g, 7.50 mmol) in CH_2_Cl_2_ (30 ml) was stirred at room temperature overnight. Then it was washed with HCl 1 N, saturated sodium bicarbonate solution and brine, dried with Na_2_SO_4_ and the solvent was evaporated to give the crude product as a white crystal which was then purified by recrystallization from ethyl acetate (Yield 47%). [mp: 415° K]. IR (KBr, cm^-1^): 1739.7 (CO β-lactam). ^1^H NMR (250 MHz, CDCl_3_) δ 3.75 (Me, s, 3H), 5.48 (H-8, d, 1H, J = 4.75), 5.63 (H-9, d, 1H, J = 4.75), 6.75–8.23 (ArH, m, 13H); ^13^C NMR (62.9 MHz, CDCl_3_) δ 55.46 (Me), 61.02 (C-8), 81.01 (C-9), 114.62–156.83 (aromatic carbons), 161.86 (CO β-lactam); GC—MS m/*z* =390 [*M*+].

### Refinement

H atoms were placed in geometrically idealized positions [*d*(C—H) = 0.93 - 0.98 Å], and refined as riding with *U*_iso_(H) = 1.2 *U*_eq_(C) for methine and aromatic H atoms or 1.5*U*_eq_(C) for methyl H atoms. The reflection 6 0 2 was omitted in final refinement.

### Crystal data

**Table:**

C_22_H_18_N_2_O_5_	*Z* = 2
*M**_r_* = 390.38	*F*(000) = 408
Triclinic, *P*1	*D*_x_ = 1.349 Mg m^−^^3^
Hall symbol: -P 1	Mo *K*α radiation, λ = 0.71073 Å
*a* = 7.7934 (4) Å	Cell parameters from 2243 reflections
*b* = 11.2813 (3) Å	θ = 2.4–26.4°
*c* = 11.8818 (2) Å	µ = 0.10 mm^−^^1^
α = 77.771 (4)°	*T* = 294 K
β = 80.948 (5)°	Block, white
γ = 71.052 (4)°	0.20 × 0.20 × 0.20 mm
*V* = 961.18 (6) Å^3^	

### Data collection

**Table:**

Rigaku R-AXIS RAPID-S diffractometer	3928 independent reflections
Radiation source: Sealed Tube	1872 reflections with *I* > 2σ(*I*)
Graphite Monochromator	*R*_int_ = 0.104
Detector resolution: 10.0000 pixels mm^-1^	θ_max_ = 26.4°, θ_min_ = 2.4°
dtprofit.ref scans	*h* = −9→8
Absorption correction: multi-scan (*SORTAV*; Blessing, 1995)	*k* = −14→14
*T*_min_ = 0.981, *T*_max_ = 0.981	*l* = −14→14
3928 measured reflections	

### Refinement

**Table:**

Refinement on *F*^2^	Secondary atom site location: difference Fourier map
Least-squares matrix: full	Hydrogen site location: inferred from neighbouring sites
*R*[*F*^2^ > 2σ(*F*^2^)] = 0.059	H-atom parameters constrained
*wR*(*F*^2^) = 0.155	*w* = 1/[σ^2^(*F*_o_^2^) + (0.0375*P*)^2^ + 0.1692*P*] where *P* = (*F*_o_^2^ + 2*F*_c_^2^)/3
*S* = 1.01	(Δ/σ)_max_ < 0.001
3927 reflections	Δρ_max_ = 0.14 e Å^−^^3^
265 parameters	Δρ_min_ = −0.15 e Å^−^^3^
0 restraints	Extinction correction: *SHELXL97* (Sheldrick, 2008), FC^*^=KFC[1+0.001XFC^2^Λ^3^/SIN(2Θ)]^-1/4^
Primary atom site location: structure-invariant direct methods	Extinction coefficient: 0.011 (2)

### Special details

**Table:**

Geometry. Bond distances, angles *etc*. have been calculated using the rounded fractional coordinates. All su's are estimated from the variances of the (full) variance-covariance matrix. The cell e.s.d.'s are taken into account in the estimation of distances, angles and torsion angles
Refinement. Refinement on *F*^2^ for ALL reflections except those flagged by the user for potential systematic errors. Weighted *R*-factors *wR* and all goodnesses of fit *S* are based on *F*^2^, conventional *R*-factors *R* are based on *F*, with *F* set to zero for negative *F*^2^. The observed criterion of *F*^2^ > σ(*F*^2^) is used only for calculating -*R*-factor-obs *etc*. and is not relevant to the choice of reflections for refinement. *R*-factors based on *F*^2^ are statistically about twice as large as those based on *F*, and *R*-factors based on ALL data will be even larger.

### Fractional atomic coordinates and isotropic or equivalent isotropic displacement parameters (Å^2^)

**Table:**

	*x*	*y*	*z*	*U*_iso_*/*U*_eq_	
O1	0.3243 (3)	0.0941 (2)	0.8790 (2)	0.0917 (11)	
O2	0.1966 (3)	0.7038 (2)	0.9341 (2)	0.0857 (10)	
O3	0.4720 (3)	0.83418 (19)	0.77456 (17)	0.0652 (8)	
O4	1.1375 (4)	0.6731 (3)	0.5285 (3)	0.1139 (15)	
O5	1.0633 (4)	0.6949 (3)	0.3578 (2)	0.1081 (12)	
N1	0.4469 (3)	0.5569 (2)	0.85135 (19)	0.0577 (9)	
N2	1.0324 (4)	0.6750 (3)	0.4631 (3)	0.0780 (12)	
C1	0.2353 (4)	0.4324 (3)	0.8902 (2)	0.0624 (11)	
C2	0.2021 (4)	0.3172 (3)	0.8986 (2)	0.0647 (12)	
C3	0.3412 (5)	0.2117 (3)	0.8731 (3)	0.0666 (12)	
C4	0.5155 (4)	0.2219 (3)	0.8378 (3)	0.0671 (12)	
C5	0.5496 (4)	0.3360 (3)	0.8297 (2)	0.0610 (11)	
C6	0.4099 (4)	0.4414 (3)	0.8569 (2)	0.0553 (11)	
C7	0.1492 (5)	0.0772 (4)	0.9103 (4)	0.1053 (17)	
C8	0.6129 (4)	0.5948 (3)	0.8068 (2)	0.0558 (11)	
C9	0.5111 (4)	0.7211 (3)	0.8556 (3)	0.0615 (11)	
C10	0.3500 (5)	0.6679 (3)	0.8888 (3)	0.0644 (11)	
C11	0.6576 (4)	0.6108 (3)	0.6781 (2)	0.0508 (10)	
C12	0.8247 (4)	0.6260 (3)	0.6300 (2)	0.0540 (11)	
C13	0.8581 (4)	0.6524 (3)	0.5124 (3)	0.0581 (11)	
C14	0.7338 (4)	0.6619 (3)	0.4389 (3)	0.0670 (11)	
C15	0.5691 (4)	0.6437 (3)	0.4861 (3)	0.0686 (14)	
C16	0.5310 (4)	0.6203 (3)	0.6038 (2)	0.0587 (11)	
C17	0.6128 (5)	0.8875 (3)	0.7357 (3)	0.0613 (11)	
C18	0.7756 (5)	0.8490 (3)	0.7842 (3)	0.0723 (14)	
C19	0.9081 (5)	0.9080 (4)	0.7365 (3)	0.0854 (17)	
C20	0.8791 (6)	1.0018 (4)	0.6426 (4)	0.0912 (17)	
C21	0.7173 (6)	1.0394 (4)	0.5945 (3)	0.0921 (19)	
C22	0.5827 (5)	0.9832 (3)	0.6415 (3)	0.0776 (14)	
H1	0.14070	0.50350	0.90690	0.0750*	
H2	0.08510	0.31120	0.92160	0.0780*	
H4	0.60970	0.15120	0.81950	0.0800*	
H5	0.66640	0.34220	0.80600	0.0730*	
H7A	0.10340	0.09920	0.98540	0.1580*	
H7B	0.15800	−0.01000	0.91200	0.1580*	
H7C	0.06770	0.13100	0.85450	0.1580*	
H8	0.71820	0.54210	0.84860	0.0670*	
H9	0.56390	0.72760	0.92280	0.0740*	
H12	0.91340	0.61820	0.67740	0.0650*	
H14	0.75970	0.68020	0.35910	0.0800*	
H15	0.48370	0.64740	0.43790	0.0820*	
H16	0.41810	0.61050	0.63460	0.0700*	
H18	0.79670	0.78430	0.84820	0.0870*	
H19	1.01790	0.88290	0.76940	0.1020*	
H20	0.96880	1.04030	0.61090	0.1100*	
H21	0.69770	1.10320	0.52980	0.1110*	
H22	0.47220	1.01030	0.60930	0.0930*	

### Atomic displacement parameters (Å^2^)

**Table:**

	*U*^11^	*U*^22^	*U*^33^	*U*^12^	*U*^13^	*U*^23^
O1	0.0952 (19)	0.0874 (18)	0.110 (2)	−0.0523 (15)	0.0269 (15)	−0.0420 (15)
O2	0.0738 (17)	0.0895 (18)	0.0920 (18)	−0.0269 (13)	0.0240 (14)	−0.0320 (14)
O3	0.0733 (15)	0.0599 (14)	0.0622 (14)	−0.0235 (11)	−0.0041 (11)	−0.0064 (11)
O4	0.0638 (18)	0.150 (3)	0.132 (3)	−0.0557 (18)	−0.0014 (17)	−0.002 (2)
O5	0.100 (2)	0.125 (2)	0.093 (2)	−0.0523 (17)	0.0469 (16)	−0.0176 (17)
N1	0.0566 (16)	0.0636 (17)	0.0527 (15)	−0.0266 (13)	0.0096 (12)	−0.0074 (13)
N2	0.066 (2)	0.073 (2)	0.089 (2)	−0.0289 (16)	0.0230 (18)	−0.0109 (18)
C1	0.061 (2)	0.068 (2)	0.0576 (19)	−0.0229 (17)	0.0046 (15)	−0.0116 (16)
C2	0.060 (2)	0.082 (2)	0.058 (2)	−0.0335 (19)	0.0078 (15)	−0.0154 (18)
C3	0.078 (2)	0.073 (2)	0.058 (2)	−0.040 (2)	0.0147 (17)	−0.0195 (17)
C4	0.071 (2)	0.074 (2)	0.060 (2)	−0.0284 (18)	0.0115 (16)	−0.0220 (17)
C5	0.059 (2)	0.072 (2)	0.0531 (18)	−0.0277 (17)	0.0127 (15)	−0.0139 (16)
C6	0.062 (2)	0.063 (2)	0.0427 (17)	−0.0280 (16)	0.0036 (14)	−0.0046 (14)
C7	0.109 (3)	0.104 (3)	0.129 (3)	−0.072 (3)	0.032 (3)	−0.045 (3)
C8	0.0566 (18)	0.064 (2)	0.0481 (17)	−0.0249 (16)	−0.0026 (14)	−0.0034 (15)
C9	0.073 (2)	0.066 (2)	0.0484 (17)	−0.0280 (17)	−0.0021 (15)	−0.0074 (16)
C10	0.067 (2)	0.071 (2)	0.0538 (19)	−0.0251 (18)	0.0074 (16)	−0.0105 (17)
C11	0.0441 (16)	0.0544 (18)	0.0525 (17)	−0.0175 (14)	0.0017 (13)	−0.0069 (14)
C12	0.0482 (18)	0.0548 (18)	0.0591 (19)	−0.0182 (14)	−0.0016 (14)	−0.0084 (15)
C13	0.0485 (18)	0.0530 (18)	0.068 (2)	−0.0188 (14)	0.0132 (15)	−0.0084 (16)
C14	0.068 (2)	0.075 (2)	0.0521 (19)	−0.0229 (18)	0.0069 (16)	−0.0058 (16)
C15	0.064 (2)	0.090 (3)	0.056 (2)	−0.0313 (19)	−0.0026 (16)	−0.0112 (18)
C16	0.0503 (18)	0.072 (2)	0.0570 (19)	−0.0273 (16)	0.0007 (14)	−0.0085 (16)
C17	0.074 (2)	0.057 (2)	0.0542 (19)	−0.0249 (17)	0.0033 (16)	−0.0115 (16)
C18	0.087 (3)	0.072 (2)	0.065 (2)	−0.036 (2)	−0.0066 (19)	−0.0086 (18)
C19	0.092 (3)	0.086 (3)	0.087 (3)	−0.044 (2)	0.000 (2)	−0.013 (2)
C20	0.109 (3)	0.081 (3)	0.089 (3)	−0.049 (3)	0.020 (3)	−0.016 (2)
C21	0.120 (4)	0.067 (3)	0.075 (3)	−0.030 (3)	0.014 (3)	0.004 (2)
C22	0.093 (3)	0.067 (2)	0.061 (2)	−0.018 (2)	−0.0005 (19)	−0.0004 (18)

### Geometric parameters (Å, °)

**Table:**

O1—C3	1.361 (4)	C15—C16	1.370 (4)
O1—C7	1.420 (5)	C17—C18	1.377 (6)
O2—C10	1.208 (5)	C17—C22	1.371 (5)
O3—C9	1.403 (4)	C18—C19	1.390 (6)
O3—C17	1.390 (5)	C19—C20	1.356 (6)
O4—N2	1.207 (5)	C20—C21	1.369 (7)
O5—N2	1.223 (4)	C21—C22	1.383 (6)
N1—C6	1.410 (4)	C1—H1	0.9300
N1—C8	1.478 (4)	C2—H2	0.9300
N1—C10	1.361 (4)	C4—H4	0.9300
N2—C13	1.471 (5)	C5—H5	0.9300
C1—C2	1.386 (5)	C7—H7A	0.9600
C1—C6	1.386 (5)	C7—H7B	0.9600
C2—C3	1.377 (5)	C7—H7C	0.9600
C3—C4	1.392 (5)	C8—H8	0.9800
C4—C5	1.378 (5)	C9—H9	0.9800
C5—C6	1.384 (5)	C12—H12	0.9300
C8—C9	1.571 (5)	C14—H14	0.9300
C8—C11	1.500 (3)	C15—H15	0.9300
C9—C10	1.525 (5)	C16—H16	0.9300
C11—C12	1.386 (5)	C18—H18	0.9300
C11—C16	1.389 (4)	C19—H19	0.9300
C12—C13	1.367 (4)	C20—H20	0.9300
C13—C14	1.368 (5)	C21—H21	0.9300
C14—C15	1.378 (5)	C22—H22	0.9300
			
O1···O3^i^	3.219 (3)	C5···H9^vii^	2.9400
O1···C17^i^	3.234 (4)	C5···H8	3.0800
O1···C22^i^	3.414 (4)	C6···H16	2.9200
O2···O3	3.155 (3)	C6···H9^vii^	2.8900
O2···C1	3.125 (4)	C7···H2	2.5500
O3···C16	3.348 (4)	C8···H18	3.0900
O3···O1^ii^	3.219 (3)	C8···H5	2.7400
O3···O2	3.155 (3)	C9···H18	2.5300
O3···N1	3.129 (3)	C10···H1	2.8000
O4···C15^iii^	3.239 (5)	C11···H5	3.0700
O4···C16^iii^	3.164 (5)	C15···H21^ix^	2.9200
O5···C12^iv^	3.414 (5)	C18···H9	2.6400
O2···H7A^v^	2.8500	C18···H12	2.9500
O2···H19^vi^	2.6800	C20···H4^ii^	3.0800
O2···H2^v^	2.5900	C21···H4^ii^	3.0800
O2···H1	2.5300	C22···H22^ix^	3.0600
O4···H12	2.4100	H1···O2	2.5300
O4···H16^iii^	2.5200	H1···C10	2.8000
O4···H15^iii^	2.6900	H2···C7	2.5500
O5···H14	2.4200	H2···H7A	2.3200
O5···H5^iv^	2.6200	H2···H7C	2.3800
N1···O3	3.129 (3)	H2···O2^v^	2.5900
N1···H16	2.5500	H4···C20^i^	3.0800
C1···O2	3.125 (4)	H4···C21^i^	3.0800
C5···C10^vii^	3.541 (4)	H5···C8	2.7400
C5···C11	3.524 (4)	H5···C11	3.0700
C6···C9^vii^	3.562 (4)	H5···H8	2.5800
C6···C16	3.432 (4)	H5···O5^iv^	2.6200
C6···C10^vii^	3.585 (5)	H7A···C2	2.7600
C7···C7^viii^	3.533 (6)	H7A···H2	2.3200
C8···C18	3.440 (5)	H7A···O2^v^	2.8500
C9···C6^vii^	3.562 (4)	H7A···H7B^viii^	2.5800
C10···C6^vii^	3.585 (5)	H7B···H7A^viii^	2.5800
C10···C5^vii^	3.541 (4)	H7C···C2	2.8000
C10···C16	3.539 (4)	H7C···H2	2.3800
C11···C17	3.234 (5)	H8···C5	3.0800
C11···C5	3.524 (4)	H8···H5	2.5800
C12···C18	3.307 (5)	H8···H12	2.5100
C12···C17	3.280 (5)	H9···C18	2.6400
C12···O5^iv^	3.414 (5)	H9···H18	2.1200
C13···C13^iv^	3.477 (5)	H9···C1^vii^	2.8600
C15···C21^ix^	3.567 (5)	H9···C2^vii^	2.8700
C15···O4^vi^	3.239 (5)	H9···C3^vii^	2.9100
C16···O3	3.348 (4)	H9···C4^vii^	2.9500
C16···C6	3.432 (4)	H9···C5^vii^	2.9400
C16···C10	3.539 (4)	H9···C6^vii^	2.8900
C16···O4^vi^	3.164 (5)	H12···O4	2.4100
C17···C12	3.280 (5)	H12···C18	2.9500
C17···O1^ii^	3.234 (4)	H12···H8	2.5100
C17···C11	3.234 (5)	H14···O5	2.4200
C18···C8	3.440 (5)	H14···C2^x^	3.0200
C18···C12	3.307 (5)	H14···C3^x^	2.8900
C21···C15^ix^	3.567 (5)	H15···O4^vi^	2.6900
C22···O1^ii^	3.414 (4)	H16···O4^vi^	2.5200
C1···H9^vii^	2.8600	H16···N1	2.5500
C2···H7C	2.8000	H16···C6	2.9200
C2···H18^vii^	2.9800	H18···C8	3.0900
C2···H14^x^	3.0200	H18···C9	2.5300
C2···H9^vii^	2.8700	H18···H9	2.1200
C2···H7A	2.7600	H18···C2^vii^	2.9800
C3···H14^x^	2.8900	H19···O2^iii^	2.6800
C3···H9^vii^	2.9100	H21···C15^ix^	2.9200
C4···H9^vii^	2.9500	H22···C22^ix^	3.0600
			
C3—O1—C7	118.6 (3)	C19—C20—C21	119.7 (4)
C9—O3—C17	116.9 (3)	C20—C21—C22	120.5 (4)
C6—N1—C8	130.9 (2)	C17—C22—C21	119.8 (4)
C6—N1—C10	133.3 (3)	C2—C1—H1	120.00
C8—N1—C10	95.8 (2)	C6—C1—H1	120.00
O4—N2—O5	123.3 (4)	C1—C2—H2	120.00
O4—N2—C13	118.4 (3)	C3—C2—H2	120.00
O5—N2—C13	118.2 (3)	C3—C4—H4	120.00
C2—C1—C6	119.9 (3)	C5—C4—H4	120.00
C1—C2—C3	120.4 (3)	C4—C5—H5	120.00
O1—C3—C2	125.4 (3)	C6—C5—H5	120.00
O1—C3—C4	115.2 (3)	O1—C7—H7A	109.00
C2—C3—C4	119.4 (3)	O1—C7—H7B	109.00
C3—C4—C5	120.5 (3)	O1—C7—H7C	109.00
C4—C5—C6	119.9 (3)	H7A—C7—H7B	109.00
N1—C6—C1	120.6 (3)	H7A—C7—H7C	110.00
N1—C6—C5	119.6 (3)	H7B—C7—H7C	109.00
C1—C6—C5	119.9 (3)	N1—C8—H8	112.00
N1—C8—C9	86.1 (2)	C9—C8—H8	112.00
N1—C8—C11	115.7 (2)	C11—C8—H8	112.00
C9—C8—C11	115.3 (3)	O3—C9—H9	113.00
O3—C9—C8	116.7 (3)	C8—C9—H9	113.00
O3—C9—C10	112.6 (3)	C10—C9—H9	113.00
C8—C9—C10	85.8 (2)	C11—C12—H12	120.00
O2—C10—N1	131.9 (3)	C13—C12—H12	120.00
O2—C10—C9	135.9 (3)	C13—C14—H14	121.00
N1—C10—C9	92.2 (3)	C15—C14—H14	121.00
C8—C11—C12	119.8 (3)	C14—C15—H15	120.00
C8—C11—C16	121.9 (3)	C16—C15—H15	120.00
C12—C11—C16	118.2 (2)	C11—C16—H16	119.00
C11—C12—C13	119.5 (3)	C15—C16—H16	119.00
N2—C13—C12	118.7 (3)	C17—C18—H18	120.00
N2—C13—C14	118.7 (3)	C19—C18—H18	120.00
C12—C13—C14	122.5 (3)	C18—C19—H19	120.00
C13—C14—C15	118.3 (3)	C20—C19—H19	120.00
C14—C15—C16	120.2 (3)	C19—C20—H20	120.00
C11—C16—C15	121.4 (3)	C21—C20—H20	120.00
O3—C17—C18	124.4 (3)	C20—C21—H21	120.00
O3—C17—C22	115.6 (3)	C22—C21—H21	120.00
C18—C17—C22	120.0 (4)	C17—C22—H22	120.00
C17—C18—C19	119.3 (3)	C21—C22—H22	120.00
C18—C19—C20	120.7 (4)		
			
C7—O1—C3—C4	−177.8 (3)	C4—C5—C6—N1	178.3 (3)
C7—O1—C3—C2	2.1 (5)	C9—C8—C11—C12	92.0 (4)
C17—O3—C9—C10	−177.7 (3)	N1—C8—C11—C16	15.1 (4)
C9—O3—C17—C22	168.1 (3)	N1—C8—C11—C12	−169.7 (3)
C17—O3—C9—C8	−80.8 (3)	N1—C8—C9—O3	−116.3 (3)
C9—O3—C17—C18	−10.9 (4)	N1—C8—C9—C10	−3.0 (2)
C6—N1—C10—O2	−4.2 (6)	C9—C8—C11—C16	−83.2 (4)
C6—N1—C8—C9	−173.9 (3)	C11—C8—C9—O3	0.4 (4)
C10—N1—C8—C9	3.4 (2)	C11—C8—C9—C10	113.7 (3)
C8—N1—C6—C1	−172.3 (2)	C8—C9—C10—N1	3.2 (2)
C8—N1—C10—O2	178.7 (4)	C8—C9—C10—O2	−179.0 (4)
C8—N1—C10—C9	−3.4 (2)	O3—C9—C10—N1	120.5 (3)
C8—N1—C6—C5	8.2 (4)	O3—C9—C10—O2	−61.8 (5)
C10—N1—C8—C11	−113.0 (3)	C12—C11—C16—C15	0.2 (5)
C6—N1—C8—C11	69.8 (4)	C16—C11—C12—C13	1.5 (5)
C10—N1—C6—C5	−168.0 (3)	C8—C11—C16—C15	175.4 (3)
C6—N1—C10—C9	173.7 (3)	C8—C11—C12—C13	−173.9 (3)
C10—N1—C6—C1	11.5 (4)	C11—C12—C13—N2	176.8 (3)
O5—N2—C13—C12	177.9 (3)	C11—C12—C13—C14	−1.5 (5)
O4—N2—C13—C14	176.2 (3)	N2—C13—C14—C15	−178.4 (3)
O5—N2—C13—C14	−3.8 (5)	C12—C13—C14—C15	−0.2 (5)
O4—N2—C13—C12	−2.2 (5)	C13—C14—C15—C16	1.8 (5)
C2—C1—C6—N1	−178.1 (2)	C14—C15—C16—C11	−1.8 (5)
C2—C1—C6—C5	1.4 (4)	O3—C17—C18—C19	178.8 (3)
C6—C1—C2—C3	−0.6 (4)	C22—C17—C18—C19	−0.1 (5)
C1—C2—C3—O1	179.6 (3)	O3—C17—C22—C21	−178.1 (3)
C1—C2—C3—C4	−0.5 (5)	C18—C17—C22—C21	1.0 (5)
O1—C3—C4—C5	−179.4 (3)	C17—C18—C19—C20	−0.6 (6)
C2—C3—C4—C5	0.8 (5)	C18—C19—C20—C21	0.4 (6)
C3—C4—C5—C6	0.1 (5)	C19—C20—C21—C22	0.5 (6)
C4—C5—C6—C1	−1.2 (4)	C20—C21—C22—C17	−1.2 (6)

Symmetry codes: (i) *x*, *y*−1, *z*; (ii) *x*, *y*+1, *z*; (iii) *x*+1, *y*, *z*; (iv) −*x*+2, −*y*+1, −*z*+1; (v) −*x*, −*y*+1, −*z*+2; (vi) *x*−1, *y*, *z*; (vii) −*x*+1, −*y*+1, −*z*+2; (viii) −*x*, −*y*, −*z*+2; (ix) −*x*+1, −*y*+2, −*z*+1; (x) −*x*+1, −*y*+1, −*z*+1.

### Hydrogen-bond geometry (Å, °)

**Table:**

Cg2 and Cg4 are the centroids of the C1–C6 and C17–C22 benzene rings, respectively.

**Table:**

*D*—H···*A*	*D*—H	H···*A*	*D*···*A*	*D*—H···*A*
C1—H1···O2	0.93	2.53	3.125 (4)	122
C2—H2···O2^v^	0.93	2.59	3.464 (4)	156
C16—H16···O4^vi^	0.93	2.52	3.164 (5)	126
C16—H16···N1	0.93	2.55	2.898 (3)	103
C4—H4···Cg4^i^	0.93	2.89	3.716 (4)	149
C9—H9···Cg2^vii^	0.98	2.55	3.463 (4)	154

Symmetry codes: (v) −*x*, −*y*+1, −*z*+2; (vi) *x*−1, *y*, *z*; (i) *x*, *y*−1, *z*; (vii) −*x*+1, −*y*+1, −*z*+2.

### Table 2 The dihedral angles (°) between the mean planes of the rings in (I)

**Table:**

	Ring 2	Ring 3	Ring 4
Ring 1	11.61 (19)	74.5 (2)	72.3 (2)
Ring 2		84.98 (15)	60.96 (16)
Ring 3			36.22 (17)

Ring 1 is the N1/C8–C10 β-lactam ring, Ring 2 is the C1–C6 benzene ring, Ring 3 is the C11–C16 benzene ring and Ring 4 is the C17–C22 benzene ring.
